# The particularities of microangiopathic and macroangiopathic complications in type 2 diabetes mellitus patients correlated with the presence or absence of type D personality

**DOI:** 10.1192/j.eurpsy.2021.661

**Published:** 2021-08-13

**Authors:** R. Kalinovic, B. Tinca, O. Neda-Stepan, M. Dinescu, C. Giurgi-Oncu, I. Enatescu, V.R. Enatescu

**Affiliations:** 1 Psychiatry I, “Pius Brinzeu” Emergency County Hospital-Psychiatric Clinic, Timisoara, Romania; 2 Psychiatry, “Victor Babes” University of Medicine and Pharmacy, Timisoara, Romania; 3 Neonatology And Childcare, “Victor Babes” University of Medicine and Pharmacy, Timisoara, Romania

**Keywords:** type D personality, depression, anxiety, diabetes mellitus

## Abstract

**Introduction:**

According to our national data based on PREDATORR study, the point prevalence of diabetes mellitus in Romania was 11.6 % in adults between 20 and 79 years old. Unequivocally, type 2 diabetes mellitus is highly correlated with psychological and personality factors.

**Objectives:**

The objective of our research was to evaluate the frequency of type D personality in patients suffering from type 2 diabetes and its influence on angiopathic complications.

**Methods:**

A cross-sectional study was conducted in 173 patients with type 2 diabetes who were self-assessed by using Beck Depression Inventory I, STAI-Y scale and DS 14 scale for detection of type D personality.

**Results:**

Both depression (p = 0.012) and state and trait anxiety (p = 0.019 and 0.023 respectively) scores were significantly higher in diabetic patients with type D personality compared with non-type D diabetic patients. Lower limb complications were more frequent in non-type D personality diabetics (p = 0.018) while diabetic retinopathy and diabetic polyneuropathy (p = 0.004 and p = 0.010 respectively).

**Conclusions:**

The presence of type D personality has a supplementary negative impact on type 2 diabetic patients’ affectivity and emotions. On the one hand, the more frequent microangiopathic complications in type D personality diabetic patients confirm that diabetes, at least in part, is an endovascular disease. On the other hand, some factors such as pro-inflammatory biomarkers may be more expressed in type 2 diabetic patients with concomitantly type D personality than those without this type of personality, leading to premature microangiopathic complications.

